# P2×_7_ Receptor in the Kidneys of Diabetic Rats Submitted to Aerobic Training or to N-Acetylcysteine Supplementation

**DOI:** 10.1371/journal.pone.0097452

**Published:** 2014-06-18

**Authors:** Adelson M. Rodrigues, Cassia T. Bergamaschi, Maria Jose S. Fernandes, Edgar J. Paredes-Gamero, Marcus V. Curi, Alice T. Ferreira, Sergio R. R. Araujo, Giovana R. Punaro, Fabiane R. Maciel, Guilherme B. Nogueira, Elisa M. S. Higa

**Affiliations:** 1 Department of Medicine, Nephrology Division, UNIFESP, Sao Paulo, Brazil; 2 Department of Physiology, Cardiovascular Division, UNIFESP, Sao Paulo, Brazil; 3 Department of Neurology/Neurosurgery, UNIFESP, Sao Paulo, Brazil; 4 Department of Biochemistry, UNIFESP, Sao Paulo, Brazil; 5 Department of Biophysics, Molecular Biology Division, UNIFESP, Sao Paulo, Brazil; 6 Department of Pathology, Investigative Pathology Division, UNIFESP, Sao Paulo, Brazil; 7 Department of Medicine, Emergency Division, UNIFESP, Sao Paulo, Brazil; Federal University of São Paulo (UNIFESP), Escola Paulista de Medicina, Brazil

## Abstract

Previous studies in our laboratory showed that N-acetylcysteine supplementation or aerobic training reduced oxidative stress and the progression of diabetic nephropathy in rats. The P2X_7_ receptor is up-regulated in pathological conditions, such as diabetes mellitus. This up-regulation is related to oxidative stress and induces tissue apoptosis or necrosis. The aim of the present study is to assess the role of P2X_7_ receptor in the kidneys of diabetic rats submitted to aerobic training or N-acetylcysteine supplementation. Diabetes was induced in male Wistar rats by streptozotocin (60 mg/kg, i.v.) and the training was done on a treadmill; N-acetylcysteine was given in the drinking water (600 mg/L). By confocal microscopy, as compared to control, the kidneys of diabetic rats showed increased P2×7 receptor expression and a higher activation in response to 2′(3′)-O-(4-benzoylbenzoyl) adenosine5'–triphosphate (specific agonist) and adenosine triphosphate (nonspecific agonist) (all p<0.05). All these alterations were reduced in diabetic rats treated with N-acetylcysteine, exercise or both. We also observed measured proteinuria and albuminuria (early marker of diabetic nephropathy) in DM groups. Lipoperoxidation was strongly correlated with P2X_7_ receptor expression, which was also correlated to NO^•^, thus associating this receptor to oxidative stress and kidney lesion. We suggest that P2X_7_ receptor inhibition associated with the maintenance of redox homeostasis could be useful as coadjuvant treatment to delay the progression of diabetic nephropathy.

## Introduction

Diabetes mellitus (DM) is an emerging global health problem; thirty percent of diabetic patients develop nephropathy, which is one of the major factors contributing to morbidity and mortality in these patients [1].

Recent studies in our laboratory showed the beneficial role of N-acetylcysteine (NAC) supplementation [2] in the oxidative stress and the progression of diabetic nephropathy. NAC is an antioxidant that acts as a free radical scavenger; it also provides cysteine, which is one of the most important sources for glutathione synthesis. Glutathione (GSH) acts intra and extracellularly as an antioxidant eliminating the reactive oxygen species (ROS), being produced naturally by the body [3].

Ji and colleagues [4] showed that blood GSH increases during prolonged exercise and would help to reduce oxidative stress; however, other studies show that training of moderate or low-intensity could reduce stress levels, directly through the glycemic control [5], [6].

Many other factors appear to contribute to the pathophysiology of diabetes, as for example, Thaning and colleagues [7] demonstrated that the vasodilatatory effect of the purinergic (P2) receptors is attenuated in these patients. These P2 receptors are formed mainly by receptors sensitive to extracellular ATP, and comprise two subfamilies, P2Y and P2X. The P2X subgroup consists of seven members (P2X_1_–7), which act as ligand-gated ion channels, mediating rapid changes in membrane permeability to cations [8]. Others studies have shown that P2X_7_ receptor (P2X_7_-R) is expressed in normal rat kidneys and in two chronic models of glomerular injury: streptozotocin-induced diabetics and in hypertensive animals [9].

The aim of the present study is to assess the P2X_7_-R in the kidneys of diabetic rats submitted to aerobic training or N-acetylcysteine supplementation.

## Methods

Male Wistar rats with 7 weeks of age and weighing between 170 to 210 g were obtained from the Central Animal Housing of *Escola Paulista de Medicina*. The protocol was approved by the Ethics Committee in Research of Universidade Federal de Sao Paulo, protocol # 0486/09. The animals were placed, in average, five/cage and they received temporary mark on the tail. All of them passed through a preselection for running; we did it because there are animals that do not adapt to a treadmill exercise routine, and these ones were allocated to the sedentary group and the others to the exercise group. From this point, they were randomly assigned to the formation of subgroups: CTL+SE (sedentary control); CTL+SE+NAC (sedentary control plus NAC); CTL+EX (trained control); CTL+EX+NAC (trained control plus NAC); DM+SE (sedentary diabetic); DM+SE+NAC (sedentary diabetic plus NAC); DM+EX (trained diabetic); DM+EX+NAC (trained diabetic plus NAC); n = 10 for each group.

### Surgical Procedure

The unillateral nephrectomy was done in all the animals in order to accelerate the development of diabetic nephropathy. The animals were anaesthetized with ketamine chloridrate (67 mg/kg,i.m.; Dopalen, Sesp, Sao Paulo, Brazil) and xylazine chloridrate (9 mg/kg,i.m.; Xilazina, Rhobiofarma, Sao Paulo, Brazil) [10], and the left kidney was removed. All the procedures were performed under sterile conditions and the animals rested for one week following the surgery [11]. After surgery, they received analgesic buprenorphine (0.05 mg/kg, sc) (Bupaq, Richterpharma, Brazil) given each 6 hours and supplemented with carprofen (5 mg/kg, sc) (Rimadyl, Oetis, Brazil) given each 12 hours [12].

### Metabolic Cages

All animals were placed in metabolic cages (Tecniplast, Italy), receiving water/NAC and chow ad libitum for 24 hours; the NAC/water and food ingestion were recorded. The metabolic cages evaluation was done before and after the experimental protocol of 8 weeks, and at the end of this period, we collected samples of 24 hours’ urine and a small aliquot of blood from the retro-orbital plexus, after 3 hours of fasting (same day); these samples were stored at −20°C.

### DM Induction

Half of the animals received a single administration of streptozotocin (STZ, 60 mg/kg BW,i.v.; Sigma-Aldrich, Sao Paulo, Brazil) dissolved in 0.1 M citrate buffer, pH4.5, and the other half received the vehicle. DM was confirmed 48 hours after induction with STZ and defined as fasting blood glucose >200 mg/dL [13].

### Physical Training

The exercise protocol started on the fifth day after induction of DM; it consisted of a moderate running on a motordriven treadmill during 60 min/day at 16 m/min, 5 days/week, during 8 weeks at no inclination (0%). The training program was preceded by an one week period of adaptation to the aerobic exercise, this adaptation was made in periods of 10, 20 and 30 minutes with speed of 10 m/min, with 2 min of interval for each time. After the adaptation week, every day the running speed was increased gradually, until the rats ran at the standard speed of 16 m/min [14].

### Euthanasia

At the end of the protocol the animals were sacrificed with a high dose of anesthetic (ketamine chloridrate at 90 mg/kg and xylazine chloridrate at 18 mg/kg, both i.m.) followed by perforation of the diaphragm.

### Nac Supplementation

The antioxidant NAC (Zambon Ltda, Sao Paulo, Brazil) was given in the drinking water, at a concentration of 600 mg/L, daily, for 8 weeks. The animals were allowed to drink it *ad libitum* [15], beginning on the fifth day of DM, and NAC daily consumption was estimated through amount of solution ingered by the animals.

### Renal Function

Plasma and urinary levels of creatinine were measured by colorimetric assay using a Labtest Creatinine kit (Centerlab Ltda, Sao Paulo, Brazil). The plasma urea concentrations were measured using a Labtest Urea CE kit (Centerlab Ltda, Sao Paulo, Brazil). The proteinuria was measured by colorimetric assay using a Sensiprot Labtest kit (Centerlab Ltda, Sao Paulo, Brazil). The albuminuria, an early marker of diabetic nephropathy, was determined by the method of radial immunodiffusion [16].

### Estimation of Lipid Peroxidation

Lipid peroxidation was estimated by the thiobarbituric acid reactive substances (TBARS) method [17], with a molar extinction coefficient of 1.56×105 cm/mol [18], [19] in plasma [20], urine [15] and kidney [21] at the end of the 8 week protocol.

### Measurement of Glutathione Enzyme Activity

Glutathione peroxidase (GPx) and glutathione reductase (GSR) activities were measured in renal tissue. For GPx activity, an enzyme-linked immunosorbent assay (ELISA) kit (E90295Ra Uscn Life Science Inc, Sao Paulo, Brazil) was used, and for GSR activity, an ELISA kit (E91314Ra Uscn Life Science Inc, Sao Paulo, Brazil) was used. The enzymatic activity was expressed as the GSR to GPx ratio [22].

### NO^•^ Measurement

The levels of NO*^•^* of the plasma, renal cortex and urinary samples were measured by chemiluminescence using a Nitric Oxide Analyzer (NOA280, Sievers Instruments Inc, CO, USA) [23], a high-sensitivity detector for measuring NO*^•^*, which is based on the following gas-phase chemiluminescent reaction between NO*^•^* and ozone:
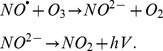



The emission of a photon from electrically excited nitrogen dioxide is in the red and near-infrared region of the spectrum, and is detected by a thermoelectrically cooled red-sensitive photomultiplier tube. The sensitivity for measurement of NO^•^ and its reaction products in liquid samples is ∼1picomole.

### Immunoblotting of Inos and Enos in the Kidneys

To determine the expression of eNOS and iNOS proteins, 40?g of total protein of each sample was separated on 8% polyacrylamide gels. The blots were then incubated with an anti-iNOS antibody (1∶200, Santa Cruz Biotechnology, CA, USA) or an anti-eNOS antibody (1∶500, BD Transduction Laboratories, CA, USA). The labeling was visualized using a secondary antibody conjugated with peroxidase for iNOS (1:5,000, Millipore Corporation, MA, USA) or eNOS (1∶500, Sigma-Aldrich, MO, USA). The bands were visualized by chemiluminescence (Amersham Pharmacia Biotech, NJ, USA) and analyzed by gel documentation (Alience4.7 Uvitec, Cambridge, United Kingdom). As a loading control, the blots were incubated with an anti-actin antibody (1∶500, Santa Cruz Biotechnology, CA, USA) which was visualized using a secondary antibody conjugated with peroxidase (1∶10,000; Sigma-Aldrich, MO, USA).

### Immunohistochemistry Analysis of P2X_7_-R

We used a method adapted to renal tissues using the primary antibody anti-P2×_7_-R (1∶200; Chemicon, CA, USA). Control slides were processed without primary antibody. The percentage of P2X_7_-R in the glomeruli was expressed as a ratio of the staining area in the glomeruli to the total area [24].

### Ca^2+^ Measurement by Confocal Microscopy

After sacrifice, the kidneys were immediately sectioned to 40µm and labeled with 10µM Fluo-4 (Invitrogen, Sao Paulo, Brazil) and pluronic 0.02% (Sigma-Aldrich, Sao Paulo, Brazil) for 1 hour. Analysis was performed by confocal laser scanning microscopy and the device was adjusted to capture the fluorescence of the focal section. The images were obtained with an argon laser (λex = 488 nm) and fluorescence was detected from 500 nm to 550 nm (LSM 780 Carl Zeiss, Jena, Germany). The activity of P2×_7_-R was assessed by the mobilization of Ca^2+^ by 100 µM BzATP (B6396, Sigma-Aldrich, Sao Paulo, Brazil) (a specific agonist to P2X_7_-R) and by 1 mM ATP (A6779, Sigma-Aldrich, Sao Paulo, Brazil) (a nonspecific agonist). The fluorescence intensity of the Fluo-4 was normalized to the basal intensity (Ft/F0), as shown in the illustrative picture, and the quantification of the brightness was expressed in arbitrary units of fluorescence (auf) [25].

### Histological Analysis

At the end of the 8-week protocol, the kidneys were removed under anesthesia. Half of each kidney was fixed in 10% formaldehyde and embedded in paraffin, sectioned to a 4 µm thickness and stained with hematoxylin–eosin (HE), and periodic acid-Schiff reagent (PAS). The analysis was carried out at a magnification of x400 and analyzed by a pathologist under blinded conditions.

### Statistical Analysis

The results are expressed as mean ±SEM and the values were compared using two-way analysis of variance (ANOVA) with Newman-Keuls post-test or non-paired T-Student test as indicated. Nonlinear regression test of polynomial type of 2nd or 3rd order was performed, and we calculated the r^2^ to analyze the curve linearity and significance was adjusted to the model. Significance was defined as p<0.05. The data were analyzed utilizing the software IBM SPSS Statistics 19.

## Results

### Metabolic Profile

At the 8^th^ week of aerobic training and/or NAC supplementation, we observed no alterations among the non-diabetic groups, opposite to the diabetic animals. The non-diabetic group, received in average, 26.6 mg/kg BW of NAC, while the diabetic groups received 264.6 mg/kg BW. When compared to CTL+SE, DM+SE rats had higher glycemia, diuresis, chow and water intake, and a reduction in body weight. NAC or aerobic training improved the metabolic profile and gain in body weight in diabetic rats; these effects were more pronounced in the animals that received both treatments (Table 1).

**Table 1:** Metabolic profile and analysis of renal function at the 8^th^ week protocol.

Parameters	CTL+SE	CTL+SE+NAC	CTL+EX	CTL+EX+NAC	DM+SE	DM+SE+NAC	DM+EX	DM+EX+NAC
Chow Intake	16.9±0.5	18.1±1.7	18.3±0.6	16.9±0.8	35.4±1.3^a^	27.4±1.8^b^	25.7±1.2^b^	26.6±0.9^b^
(mg/24 hr)								
Liquid Intake	20.0±1.36	20.3±1.3	17.3±0.7	15.7±0.9	155.1±7.8^a^	117.7±9.6^b^	117.0±5.3^b^	109.2±5.5^b^
(mL/24 hr)								
Diuresis	13.4±0.4	16.2±1.6	13.7±0.5	13.8±0.7	143.4±8.3^a^	95.3±2.5^b^	87.5±5.2^b^	91.3±2.6^b^
(mL/24 hr)								
Body Weight	424.4±6.3	421.4±10.6	406.4±11.0	388.4±4.2	186.4±6.6^a^	241.2±10.4^b^	271.9±10.5^b,c^	277.0±7.4^b,c^
(g)								
Plasma Creatinine	0.85±0.05	0.91±0.07	0.92±0.06	0.91±0.1	1.81±0.07^a^	1.13±0.1^b^	0.85±0.04^b,c^	0.91±0.03^b,c^
(mg/dL)								
Creatinine Clearance	1.07±0.08	1.01±0.07	1.3±0.07	1.4±0.17	0.5±0.06^a^	1.1±0.1^b^	1.4±0.17^b^	1.4±0.23^b^
(mL/min)								
Plasma Urea	44.7±2.4	44.2±2.7	34.3±1.8	41.4±4.3	106.3±20.5^a^	77. 6±7.6^b^	77.1±9.0^b^	67.8±4.4^b^
(mg/dL)								
Blood Glucose	105.0±2.8	107.9±2.7	99.1±2.4	105.8±1.8	526.1±15.7^a^	399.6±10.6^b^	322.7±6.8^b,c^	294.3±9.0^b,c,d^
(mg/dL)								
Albuminuria	2.06±0.44	3.24±0.7	4.59±0.7	3.3±0.36	26.98±1.75^a^	12.53±0.65^b^	12.30±1.78^b^	10.84±0.43^b^
(mg/24 hr)								
Proteinuria	9.8±0.9	8.8±0.5	12.7±0.85	13.0±0.9	38.0±2.7^a^	23.9±1.6^b^	13.5±0.5^b,c^	12.6±1.0^b,c^
(mg/24 hr)								

Results are represented as mean ±SEM. Albuminuria with n = 5 and all others parameters with n = 10. two-way ANOVA with Newman-Keuls post-test; *p*<0.05: ^a^ vs. CTL+SE; ^b^ vs. DM+SE; ^c^ vs. DM+SE+NAC; ^d^ vs. DM+EX.

CTL+SE, sedentary control; CTL+SE+NAC, sedentary control plus NAC; CTL+EX, training control; CTL+EX+NAC, training control plus NAC; DM+SE, sedentary diabetic; DM+SE+NAC, sedentary diabetic plus NAC; DM+EX, training diabetic; DM+EX+NAC, training diabetic plus NAC.

### Renal Function

At the end of the 8^th^ week, the renal function of normoglycemic rats submitted to NAC or training was unchanged. The diabetic animals showed significant increases in plasma urea and creatinine, with a reduction in creatinine clearance and increase in proteinuria and albuminuria. NAC administration and/or training improved these parameters of renal function (Table 1).

### Estimation of Lipid Peroxidation

**Figure 1:**
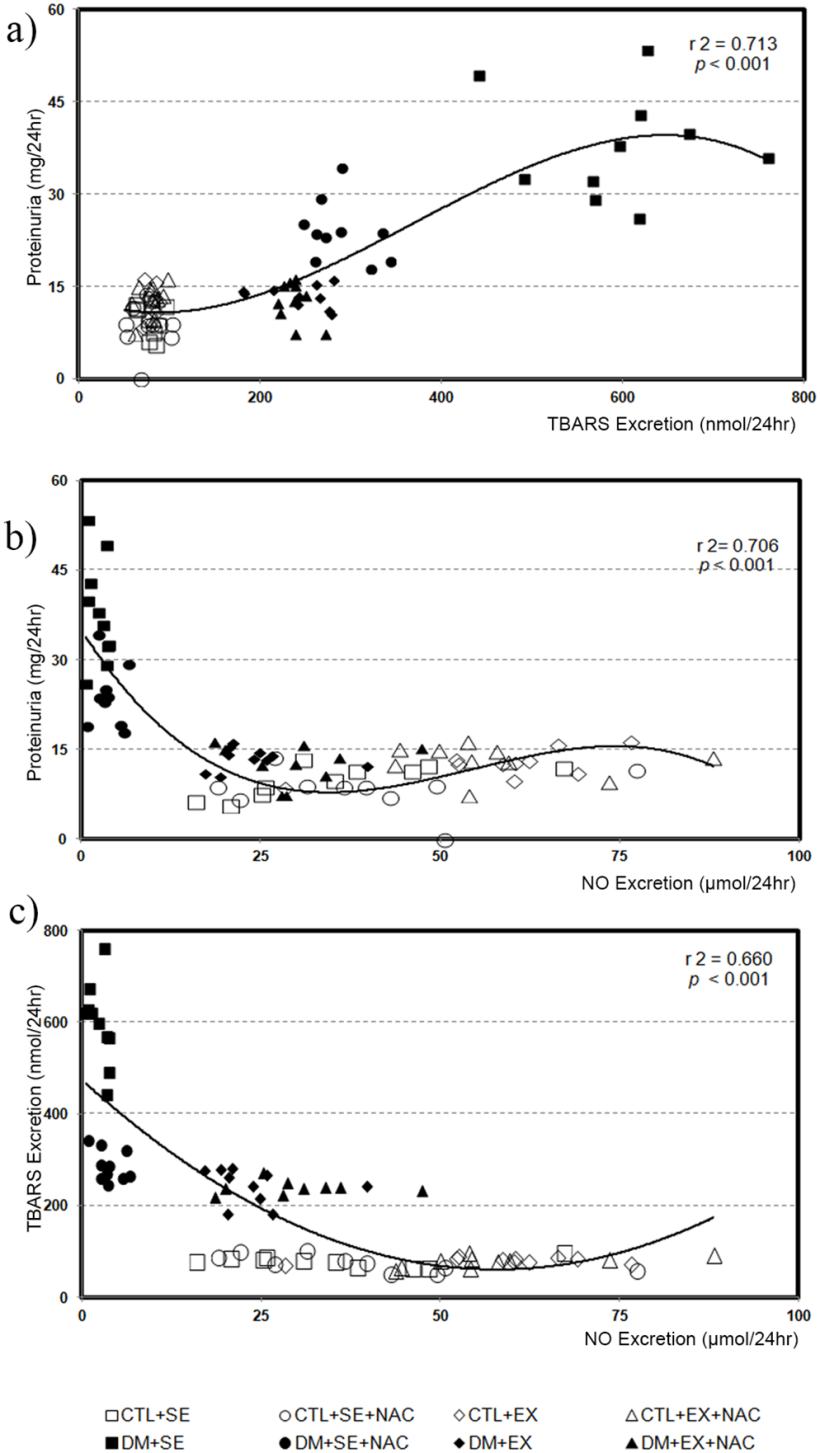
Oxidative stress and diabetic nephropathy at the 8^th^ week of protocol. Nonlinear regression oxidative stress, NO^•^ and proteinuria, n = 10 for all groups. a) TBARS urinary excretion and proteinuria are related (*p*<0.001 and r^2^ = 0.713); b) NO^•^ urinary excretion and proteinuria are also linked (*p*<0.001 and r^2^ = 0.706); c) TBARS and NO^•^ urinary excretion are dependent (*p*<0.001 and r^2^ = 0.660) (*n* = 10). CTL+SE, sedentary control; CTL+SE+NAC, sedentary control plus NAC; CTL+EX, training control; CTL+EX+NAC, training control plus NAC; DM+SE, sedentary diabetic; DM+SE+NAC, sedentary diabetic plus NAC; DM+EX, training diabetic; DM+EX+NAC, training diabetic plus NAC; TBARS, thiobarbituric acid reactive substances; NO^•^, nitric oxide.

At the end of the 8^th^ week, the DM+SE rats had the higher levels of TBARS compared to the CTL+SE rats in the plasma (nmol/mL) and urine (nmol/24 hr) (8.9±0.4 vs. 5.8±0.23; 596.6±28.2 vs. 78.1±3.5; *p*<0.05; respectively) (Table 2). Plasma and urine TBARS were reduced following NAC (5.1±0.4; 288.7±10.7; respectively) or after exercise (4.2±0.2; 242.9±12.1; respectively) and when administered together, the treatments resulted in additional improvements in plasma (2.9±0.25) and urine (237.1±4.8) (Table 2). DM+SE had a high estimated value of lipidic peroxidation in the renal cortex compared to CTL+SE rats (1.2±0.2 vs. 0.6±0.1). This value was significantly reduced in the group treated with NAC (0.6±0.1), in trained animals (0.6±0.2) or in animals that received both treatments (0.7±0.1) (Table 2). We analyzed TBARS excretion in relation to the development of proteinuria, a marker for diabetic nephropathy. According to the nonlinear regression, proteinuria and TBARS excretion were significantly related (p<0.001 and r2 = 0.713) (Figure 1a).

**Table 2:** xidative stress analysis at the 8th week protocol.

Parameters	CTL+SE	CTL+SE+NAC	CTL+EX	CTL+EX+NAC	DM+SE	DM+SE+NAC	DM+EX	DM+EX+NAC
Plasma TBARS	5.8±0.23	4.8±0.4	4.9±0.2	4.7±0.1	8.9±0.4 ^a^	5.1±0.4 ^b^	4.2±0.2 ^b^	2.9±0.25 ^b,c,d^
(nmol/mL)								
TBARS Excretion	78.1±3.5	75.5±5.8	80.6±1.9	78.8±4.1	596.6±28.2 ^a^	288.7±10.7 ^b^	242.9±12.1 ^b,c^	237.1±4.8 ^b,c^
(nmol/24 hr)								
Renal TBARS	0.6±0.1	0.6±0.1	0.6±0.05	0.8±0.1	1.2±0.2 ^a^	0.6±0.1 ^b^	0.6±0.2 ^b^	0.7±0.1 ^b^
(nmol/mg protein)								
GSR/GPx	3.8±0.7	5.2±0.3	11.8±1.9 ^a^	11.1±0.9 ^a^	1.8±0.2	4.6±0.5	6.2±1.5 ^b^	10.9±2.1 ^b,c,d^
Plasma NO	46.3±3.1	50.2±2.9	61.8±5.5	63.1±6.8	60.5±2.6	60.5±2.4	66.7±6.8	71.3±3.3
(µmol/L)								
NO^•^ Excretion	35.4±4.8	39.6±5.4	58.7±4.1 ^a^	57.9±4.3 ^a^	2.4±0.4 ^a^	3.8±0.6	23.9±2.0 ^b,c^	29.9±2.6 ^b,c^
(µmol/24 hr)								
Renal Tissue NO	931.3±42.6	1230±133.7	1459±77.4	1468±291.6	1082±42.6	1747±173.5 ^b^	2510±425.6 ^b^	2490±228.0 ^b^
(µmol/mg protein)								

Results are represented as mean ±SEM. All parameters with n = 10. two-way ANOVA with Newman-Keuls post-test; *p*<0.05: ^a^ vs. CTL+SE; ^b^ vs. DM+SE; ^c^ vs. DM+SE+NAC;^ d^ vs. DM+EX.

CTL+SE, sedentary control; CTL+SE+NAC, sedentary control plus NAC; CTL+EX, training control; CTL+EX+NAC, training control plus NAC; DM+SE, sedentary diabetic; DM+SE+NAC, sedentary diabetic plus NAC; DM+EX, training diabetic; DM+EX+NAC, training diabetic plus NAC; TBARS, thiobarbituric acid reactive substances; GSR, glutathione reductase enzyme; GPx, glutathione peroxidase enzyme; NO^•^, nitric oxide.

### Measurement of Glutathione Enzyme Activity

We analyzed the enzymatic activity by assessing the ratio of glutathione reductase (GSR) and glutathione peroxidase (GPx) in renal tissue, at the end of the 8^th^ week (Table 2). We observed that in CTL+EX and CTL+EX+NAC rats, the GSR to GPx ratio was significantly increased compared to CTL+SE rats (11.8±1.9; 11.1±0.9 vs. 3.8±0.7). These values were reduced in the DM+SE (1.8±0.2) compared to CTL+SE animals, although not significantly. DM+SE+NAC (4.6±0.5; NS), DM+EX (6.2±1.5; *p*<0.05) and DM+EX+NAC (10.9±2.1; *p*<0.05) had an increase in this ratio, when compared to DM+SE rats (Table 2).

### Measurement of NO^•^

After the 8th week of the protocol the plasma concentrations of NO^•^ had no difference among all groups; however, we observed that NO^•^ excretion was significantly increased in CTL+EX and CTL+EX+NAC compared to CTL+SE animals (58.7±4.1; 57.9±4.3 vs. 35.4±4.8; respectively). Compared to CTL+SE, NO^•^ excretion was reduced in DM+SE (2.4±0.4) (*p*<0.05); In DM+SE+NAC it was unchanged (3.8±0.6), and significantly increased in DM+EX (23.9±2.0) and DM+EX+NAC animals (29.9±2.6) when compared to DM+SE (Table 2). Using regression, we found that the variables NO^•^ excretion and proteinuria were related (*p*<0.001). Specifically, the increase in excreted NO^•^ was linked with a reduction in proteinuria r^2^ = 0.706 (Figure 1b). We also used regression test to assess the relation between TBARS and NO^•^ excretion and we observed a dependence (*p*<0.001 and r^2^ = 0.660) between these variables (Figure 1c).

Normoglycemic groups showed no changes in renal NO^•^ and these levels were similar in the DM+SE and CTL+SE group (1,082±42.6 vs. 931.3±42.6). There were significant increases in renal NO^•^ levels in DM+SE+NAC (1,747±173.5), DM+EX (2,510±425.6) and DM+EX+NAC (2,490±228.0) compared to DM+SE animals (Table 2).

### Western Blot of NOS in the Renal Cortex

**Figure 2:**
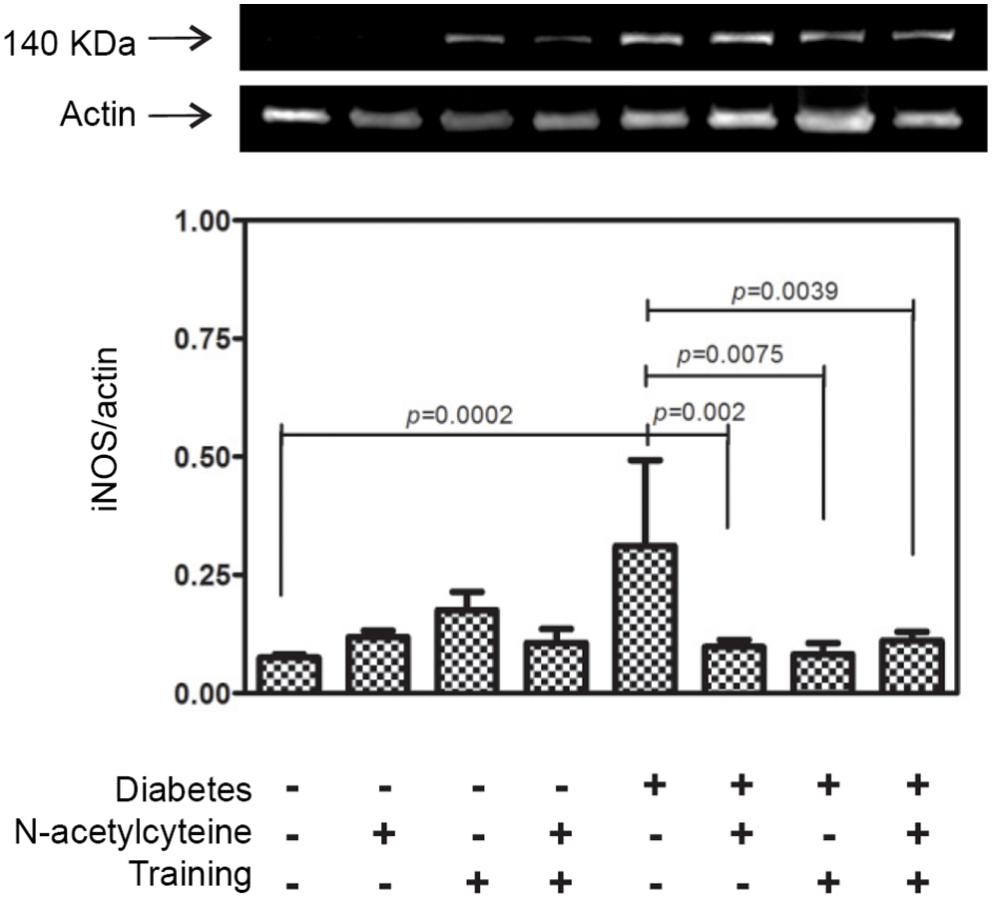
Western blot analysis of iNOS in samples of renal cortex at the 8^th^ week of protocol. Non-paired T- Student test; significance for *p*<0.05. iNOS, inducible nitric oxide synthase.

We observed that there was no difference in iNOS among the control animals. However, in diabetic animals, the DM+SE had higher values when compared to CTL+SE animals (0.31±0.18 vs. 0.075±0.007; p<0.0002). The other diabetic groups showed a reduction in these values after NAC supplementation (0.097±0.015; *p*<0.002), aerobic training (0.082±0.024; *p*<0.0075) or both (0.110±0.019; *p*<0.0039), when compared to DM+SE animals (Figure 2).

**Figure 3:**
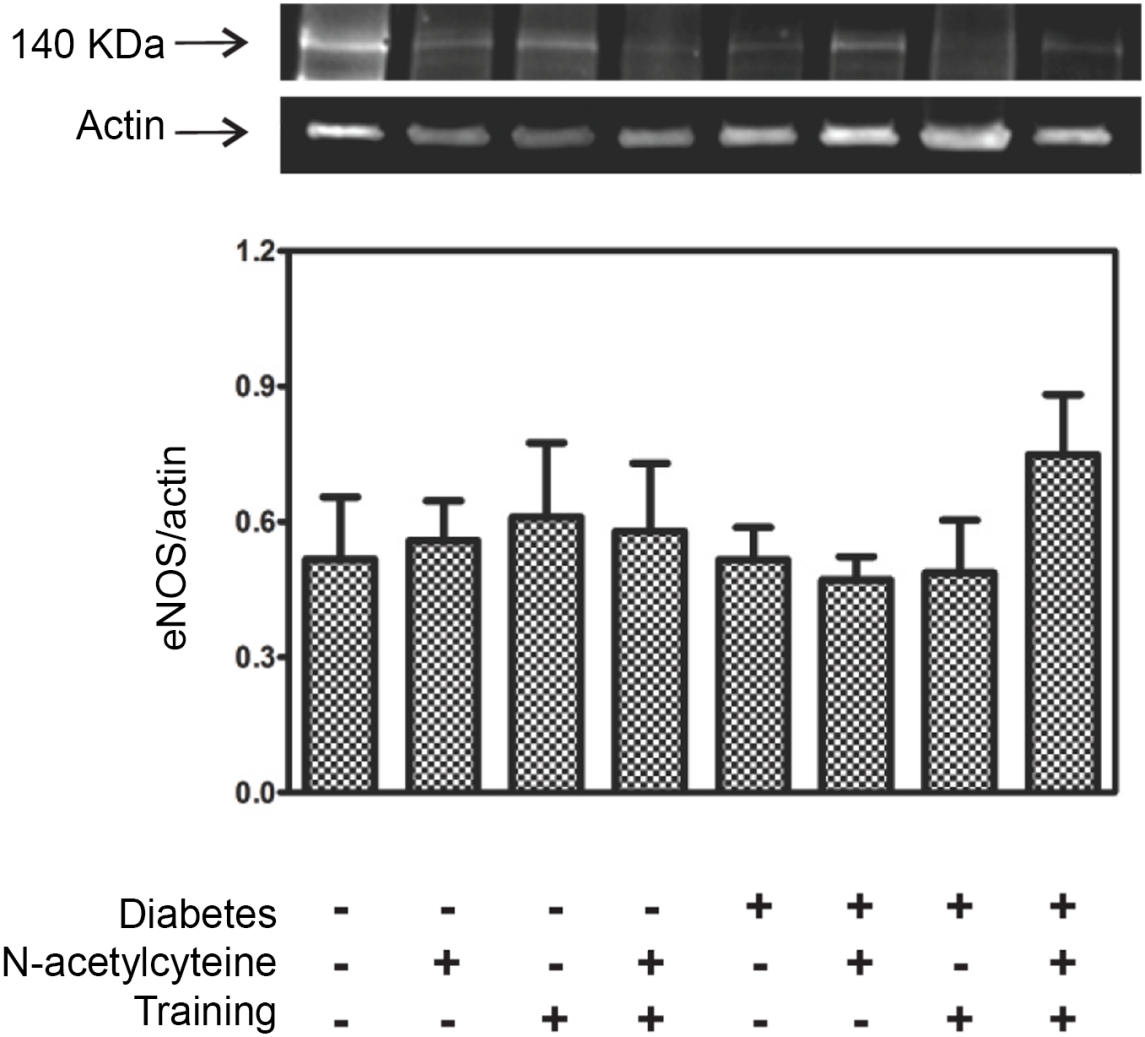
Western blot analysis of eNOS in samples of renal cortex at the 8^th^ week of protocol. eNOS, endothelial nitric oxide synthase.

The analysis of the protein levels of eNOS showed that among the control animals, there was no difference between the groups. In contrast, in diabetic animals, we observed that levels of eNOS in the DM+EX+NAC was increased compared to DM+SE animals, but it was not statistically significant (0.75±0.13 vs. 0.56±0.088) (Figure 3).

### Immunohistochemical Analysis of P2×_7_-R

**Figure 4:**
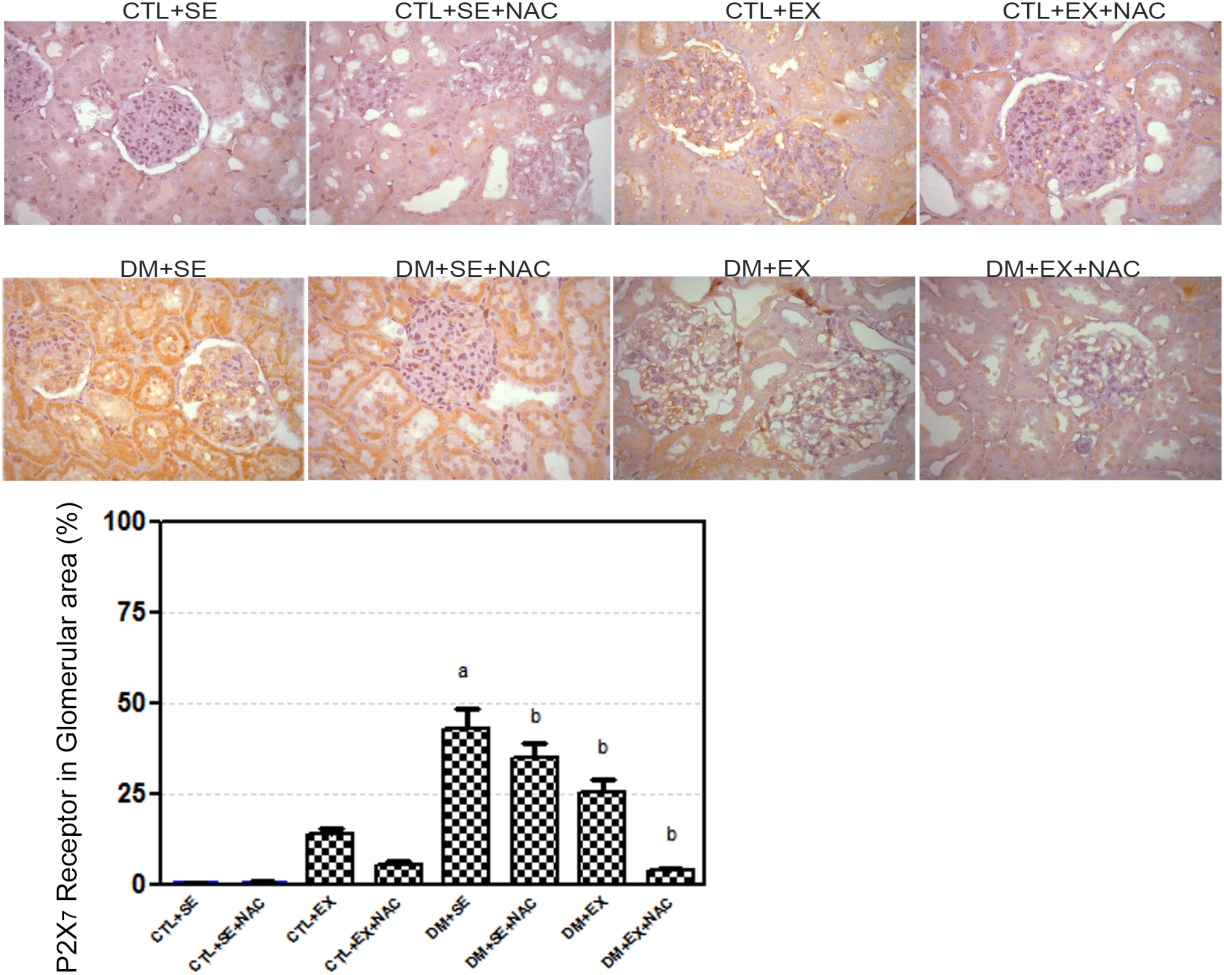
Immunohistochemistry analysis of P2×_7_-R in the kidney at the 8^th^ week of protocol. Two-way ANOVA with Newman-Keuls post-test. *p*<0.05: ^a^ vs. CTL+SE; ^b^ vs. DM+SE; ^c^ vs. DM+SE+NAC; ^d^ vs. DM+EX. CTL+SE, sedentary control; CTL+SE+NAC, sedentary control plus NAC; CTL+EX, training control; CTL+EX+NAC, training control plus NAC; DM+SE, sedentary diabetic; DM+SE+NAC, sedentary diabetic plus NAC; DM+EX, training diabetic; DM+EX+NAC, training diabetic plus NAC; P2X7-R, P2X7 receptor.

In all non-diabetic groups, there were no differences in renal tissue in P2×_7_-R, as determined by immunohistochemistry. In the diabetic animals P2×_7_-R had the highest expression in the DM+SE compared to CTL+SE rats (43.25±5.45%; *p*<0.05). The diabetic rats treated with NAC showed attenuated P2×_7_-R expression (34.87±4.34%; *p*<0.05), which was reduced to a greater extent in the trained group (25.60±3.44%; *p*<0.05), and the combination of both resulted in an additional reduction (3.85±0.56%; *p*<0.05) when compared to DM+SE (Figure 4).

### Intracellular Calcium Measurement and P2×_7_-R in Kidneys by Confocal Microscopy

**Figure 5:**
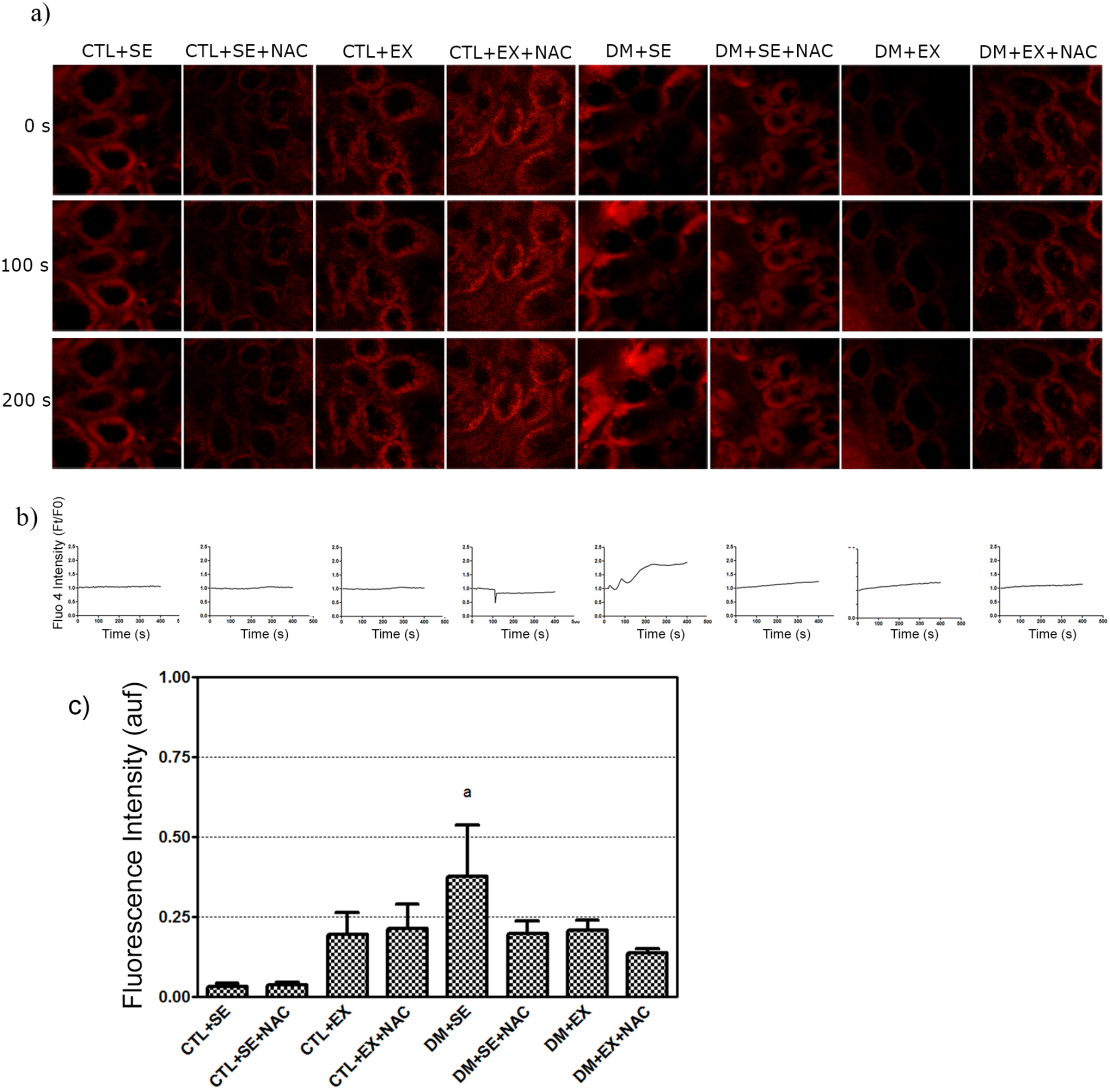
Confocal analysis of P2×_7_-R in the kidney at the 8^th^ week of protocol. a) Intracellular calcium concentration by confocal microscopy. Intensity was quantified using pseudocolor image according to fluorescence intensity by Fluo-4; these images showed the calcium mobilization in renal tissue when exposed to ATP 1 mM. Micrographies were obtained with x400 of magnification. b) ­Graphics of the calcium dynamics in relation to basal fluorescence. c) Quantification after the nonspecific agonist. Each group with n = 5, Two-way ANOVA with Newman-Keuls post-test. p<0.05: a vs. CTL+SE; b vs. DM+SE. CTL+SE, sedentary control; CTL+SE+NAC, sedentary control plus NAC; CTL+EX, training control; CTL+EX+NAC, training control plus NAC; DM+SE, sedentary diabetic; DM+SE+NAC, sedentary diabetic plus NAC; DM+EX, training diabetic; DM+EX+NAC, training diabetic plus NAC; auf, arbitrary unit fluorescence.

Longitudinal sections of the kidneys were incubated with a Fluo-4 fluorescence probe that increases the intensity of fluorescence when binding to calcium (Ca^2+^). When 1 mM ATP, a nonspecific agonist for P2X and P2Y, was used, we observed that non-diabetic groups did not show differences in Ca^2+^ influx. The DM+SE animals had the strongest response to ATP (0.38±0.16 vs. 0.034±0.01; *p*<0.05) and this influx was reduced in DM+SE+NAC, DM+EX and DM+EX+NAC, when compared to DM+SE, although not significantly (Figure 5a, 5b and 5c).

**Figure 6:**
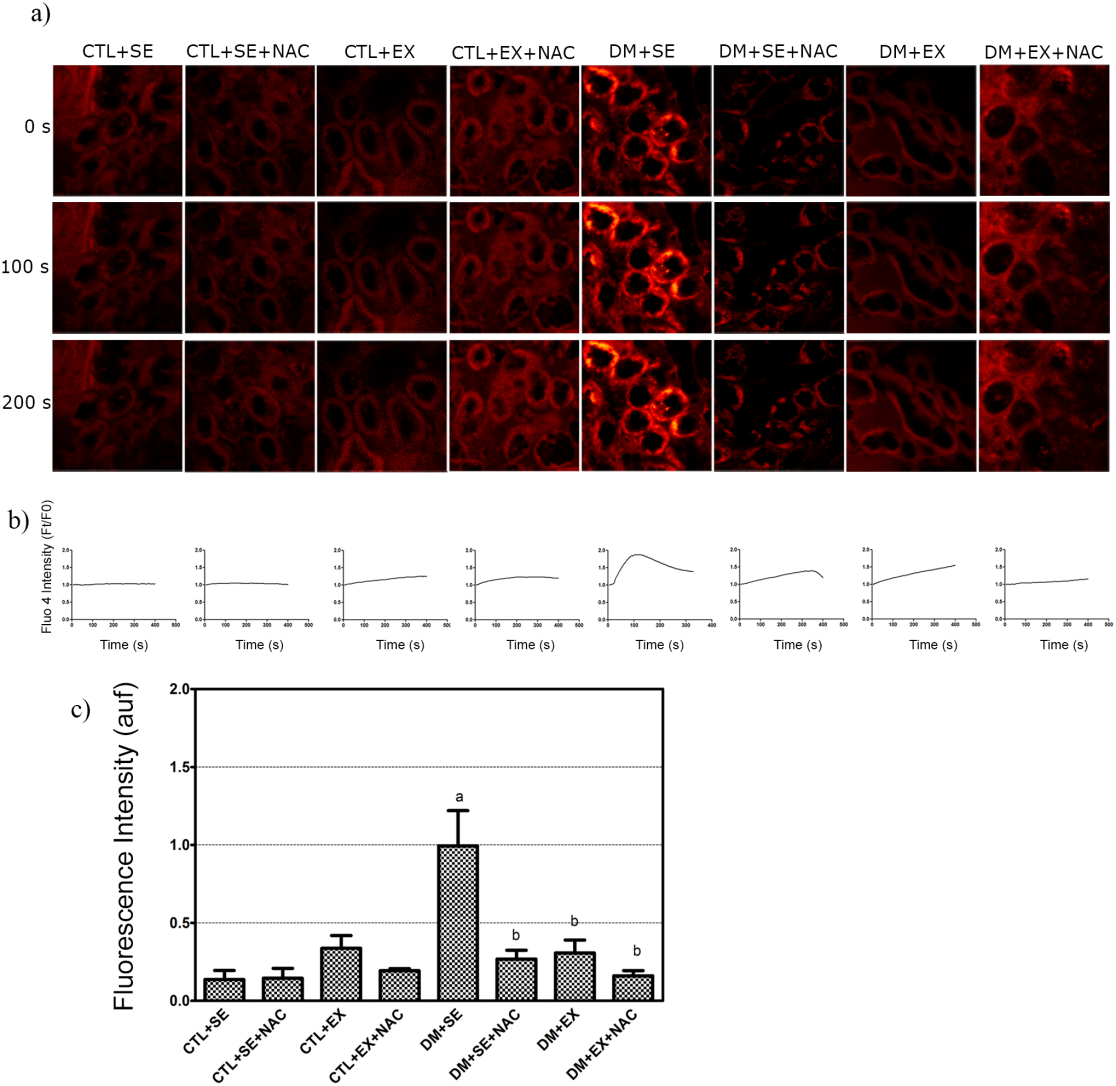
Confocal analysis of P2×_7_-R in the kidney at the 8^th^ week of protocol. a) Intracellular calcium concentration by confocal microscopy. Intensity was quantified using pseudocolor image according to fluorescence intensity by Fluo-4; these images showed the calcium mobilization in renal tissue when exposed to BzATP 100µM. Micrographies were obtained with x400 of magnification. b) ­Graphics of the calcium mobilization in relation to basal fluorescence. c) Quantification after the specific agonist. Each group with n = 5, Two-way ANOVA with Newman-Keuls post-test. p<0.05: a vs. CTL+SE; b vs. DM+SE. CTL+SE, sedentary control; CTL+SE+NAC, sedentary control plus NAC; CTL+EX, training control; CTL+EX+NAC, training control plus NAC; DM+SE, sedentary diabetic; DM+SE+NAC, sedentary diabetic plus NAC; DM+EX, training diabetic; DM+EX+NAC, training diabetic plus NAC; auf, arbitrary unit of fluorescence.

When we administered 100 µM of BzATP, a preferential agonist of P2×_7_-R, we observed again that there was no difference among the non-diabetic groups in the Ca^2+^ influx. In diabetic animals, the DM+SE group showed increased fluorescence compared to CTL+SE animals (1.0±0.23 vs. 0.14±0.06; *p*<0.05). In the DM+SE+NAC (0.27±0.06), DM+EX (0.31±0.08) and DM+EX+NAC (0.17±0.04) groups, this fluorescence intensity was significantly reduced compared to the DM+SE group (Figure 6a, 6b and 6c).

### P2X_7_-R and Diabetic Nephropathy

**Figure 7:**
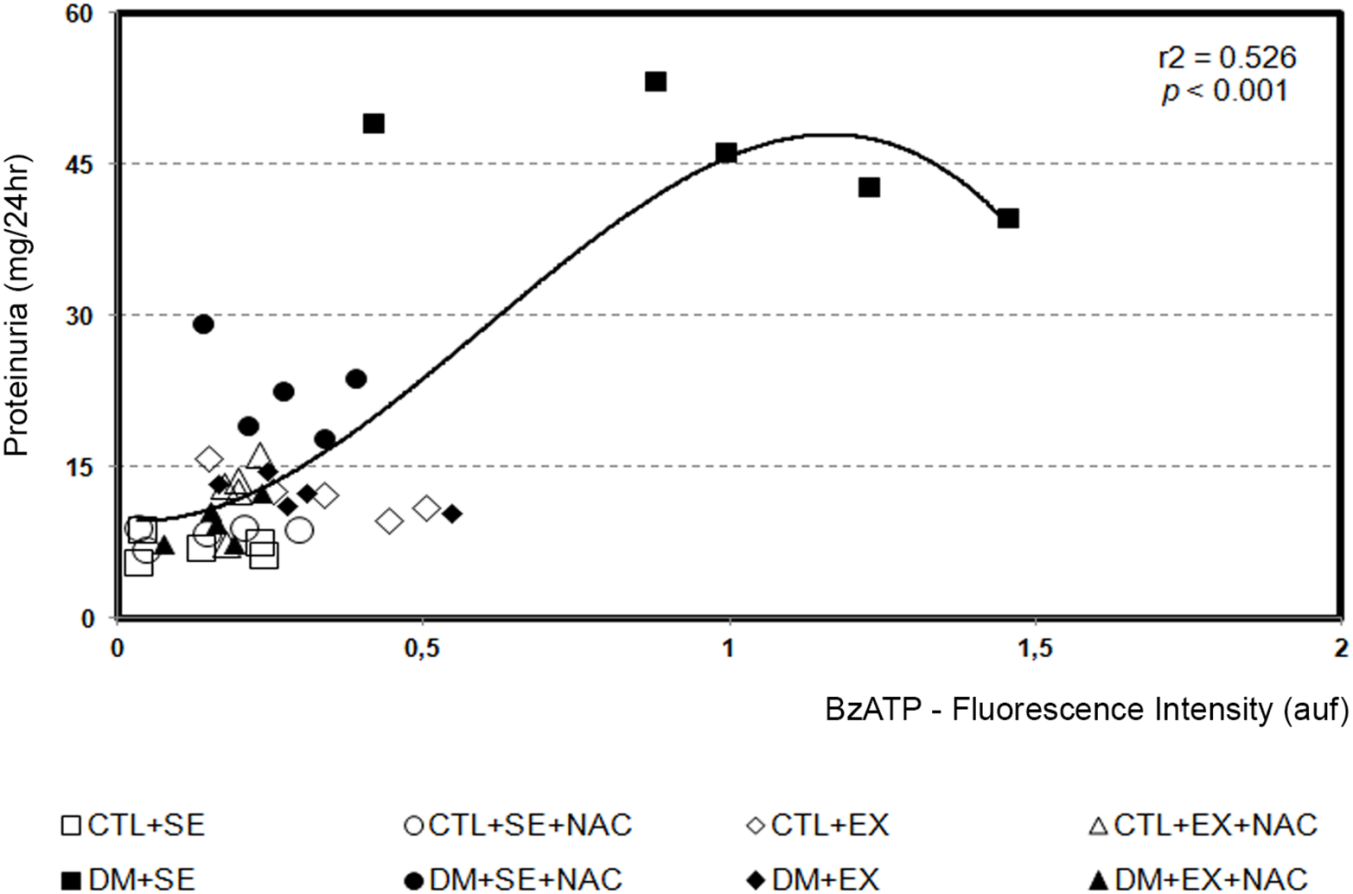
P2X_7_-R activity and diabetic nephropathy at the 8th week of protocol. Nonlinear regression between the oxidative stress, P2X_7_-R and proteinuria. calcium dynamic by BzATP and proteinuria are also related (*p*<0.001 and r^2^ = 0.526) (*n* = 5). CTL+SE, sedentary control; CTL+SE+NAC, sedentary control plus NAC; CTL+EX, training control; CTL+EX+NAC, training control plus NAC; DM+SE, sedentary diabetic; DM+SE+NAC, sedentary diabetic plus NAC; DM+EX, training diabetic; DM+EX+NAC, training diabetic plus NAC; TBARS, thiobarbituric acid reactive substances; NO^•^, nitric oxide; BzATP, 2′(3′)-O-(4-benzoylbenzoyl) adenosine 5′ –triphosphate; P2X_7_-R, P2X_7_ receptor.

We observed a correlation between P2×_7_-R activity, as determined by BzATP, and proteinuria (*p*<0.001 and r^2^ = 0.526) (Figure 7). P2X_7_-R was also related with TBARS (*p*<0.001 and r^2^ = 0.783) and NO^•^ (*p*<0.001 and r^2^ = 0.449).

### Histological Analysis

**Figure 8:**
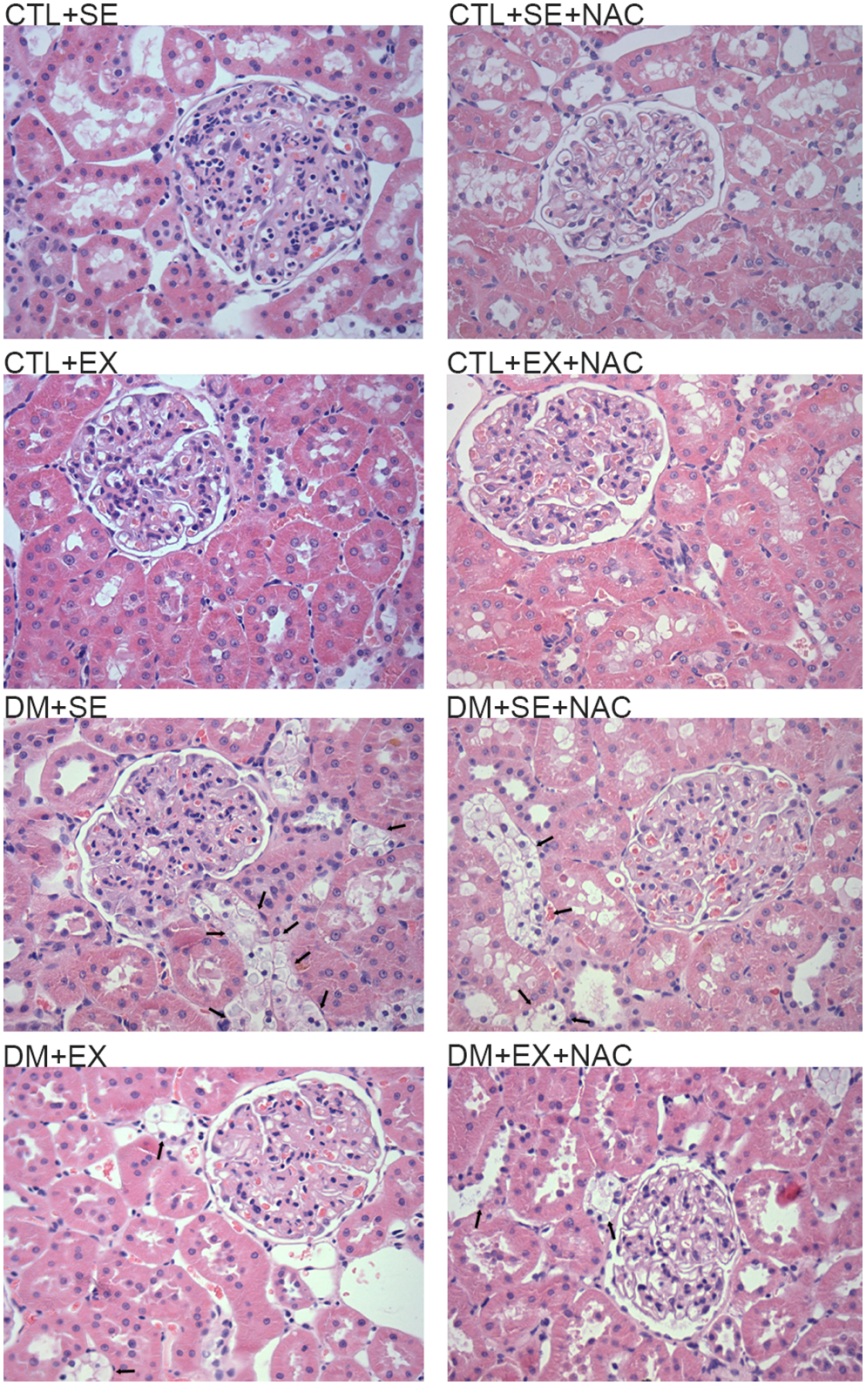
HE staining of renal tissue at the 8^th^ week of protocol. The black arrows on the micrographies show that tubular vacuolization, in DM+SE were present at proportion of 10∶12. In DM+SE+NAC this proportion was 6∶12, DM+EX and DM+EX+NAC had 04:12. Magnification of x400. CTL+SE, sedentary control; CTL+SE+NAC, sedentary control plus NAC; CTL+EX, training control; CTL+EX+NAC, training control plus NAC; DM+SE, sedentary diabetic; DM+SE+NAC, sedentary diabetic plus NAC; DM+EX, training diabetic; DM+EX+NAC, training diabetic plus NAC; HE, hematoxylin and eosin.

**Figure 9:**
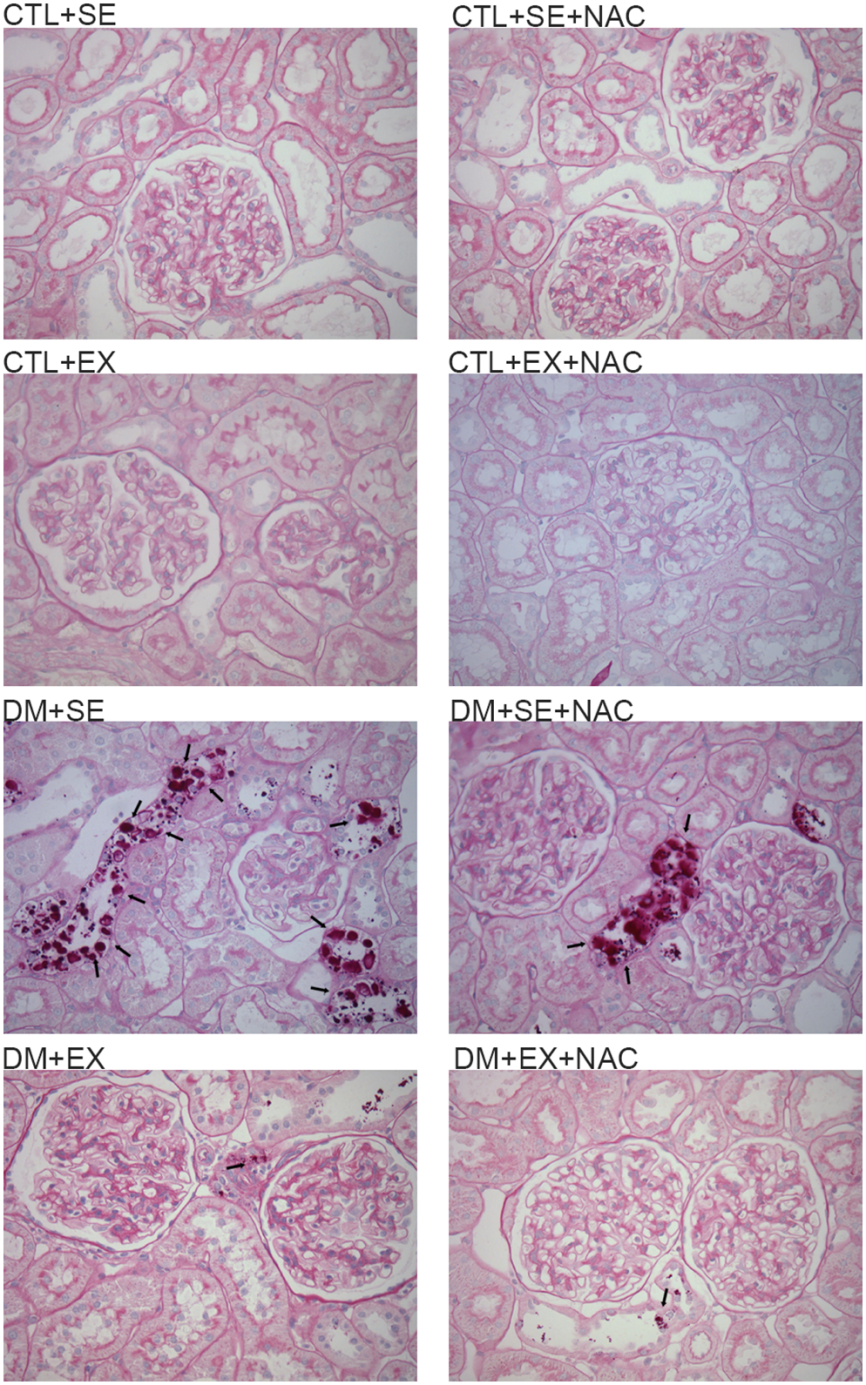
PAS staining of renal tissue at the 8^th^ week of protocol. The black arrows on the micrographies show glycosidic degeneration in the tubules. DM+SE presented these alterations in medulla and renal cortex. There was a reduction in DM+SE+NAC and DM+EX with incidence only in the renal cortex; the DM+EX+NAC was the less affected. Magnification with x400. CTL+SE, sedentary control; CTL+SE+NAC, sedentary control plus NAC; CTL+EX, training control; CTL+EX+NAC, training control plus NAC; DM+SE, sedentary diabetic; DM+SE+NAC, sedentary diabetic plus NAC; DM+EX, training diabetic; DM+EX+NAC, training diabetic plus NAC; PAS, periodic acid-Schiff.

By HE staining the diabetic groups showed serious histological modifications. Specifically, the DM+SE animals had alterations in mesangial cells, i.e., increasing mesangium matrix, and frequent tubular vacuolization and degeneration, mainly in the distal tubule (10∶12). In contrast, DM+SE+NAC animals only showed tubular vacuolization (7∶12), and this phenomenon was strongly reduced in DM+EX and DM+EX+NAC animals (4∶12 for both) (Figure 8). The PAS stain showed no alterations among the control groups. On the other hand, we observed that in DM+SE animals, there was an intense glycosidic degeneration, which was attenuated by NAC administration or aerobic training; this reduction was most evident when the treatments were combined (Figure 9).

## Discussion

Previous studies with diabetic animals, in our laboratory, showed that NAC promoted renoprotective effects, with substantial improvements in renal function and control of oxidative stress, increasing NO^•^ bioavailability [2]. These results were similar to those obtained with aerobic training, which reestablished the redox balance, delayed endothelial dysfunction and improved NO^•^ levels, with preservation of the glomerular structures and reduction of albuminuria and renal injury [14]. Therefore, the strategies above could be useful as adjuvant treatments against diabetic nephropathy. In the present study, we observed that the combination of NAC and aerobic training produced significant improvements in metabolic parameters and controlled oxidative stress (NO^•^, iNOS, TBARS and glutathione). Notably, we observed that, in the renal tissues of diabetic animals, NAC supplementation and/or aerobic training resulted in the reduced expression and activity of P2×_7_-R.

The main effect of NAC supplementation and aerobic exercise was on hyperglycemia, which is characteristic of DM, and stimulates the production of oxidative stress [26]. NAC is a potent antioxidant that decreases the ROS mainly by stimulating glutathione synthesis [27]. The exercises increase the glutathione levels [28], and in addition, decrease the hyperglycemia [29], consequently reducing the oxidative stress. Afolayan and Sunmonu [30] showed in theirs studies that glutathione peroxidase and glutathione reductase are significantly reduced in diabetic animals. These antioxidant enzymes were shown to be increased by a moderate training [31], and such changes were more intense when the participants received antioxidant supplementation in addition to exercise [32]. All of these studies corroborate with our findings and suggest that the treatments reduced lipoperoxidation and increased the GSH production.

The action of the glutathione enzymes is relevant for the maintenance of redox homeostasis, which is important for the preservation of many organs, including the kidney.

The oxidative stress from diabetes causes damage in to renal tissue which is evidente with markers such as microalbuminuria. Although there is a study showing the albuminuria resulting from exercises [33], the majority of the investigations show the opposite, i.e. exercises had a renoprotective function with decrease of albuminuria. These studies corroborate with our findings, in other words, the diabetic animals have the highest albuminuria and proteinuria and with NAC supplementation and/or aerobic training there were significant reduction in both values. We observed that proteinuria in diabetic rats was strongly linked to lipid peroxidation and NO^•^. i.e. the diabetic animals that had the highest proteinuria had the highest lipoperoxidation and the lowest NO^•^ levels.

The diabetic animals that received NAC supplementation or exercises showed a reduction in oxidative stress and an increase in NO^•^ bioavailability. Notably, when both treatments were given in combination, the best control of redox homeostasis was achieved.

Our findings are also in agreement with some papers that have demonstrated the beneficial effects of aerobic training in reducing oxidative stress [34] or increasing the antioxidant defense [35]. However, in these studies, the authors observed that this protective action of exercises was eliminated when the training was performed at a great workload.

We also analyzed the expression of NOS isoforms. Initially, we analyzed the inducible enzyme, which is synthesized in response to external stimuli or by pathologies as the diabetes [36]; however, this iNOS production look like to be modulate by oxidative stress, thus the action of antioxidants could reduce its expression [10]. Likewise the administration of antioxidantes, the use of protocols with aerobic training also showed decrease of iNOS [37]. All these studies corroborate with our findings that iNOS protein expression was increased in diabetic animals and it was significantly reduced by NAC supplementation and/or aerobic training.

The eNOS isoform is a constitutive enzyme that, in our protocol, did not show altered expression in the control groups after NAC supplementation or aerobic exercise. However, among the diabetic groups, we observed a slight increase in eNOS in the animals that received NAC and exercises. This effect could be due to the improved preservation of the endothelial cells against the damages from DM. Grutzmacher and colleagues [38] showed that the endothelial injury from DM begins in the early phase of the disease. With diabetes progression, the oxidative stress becomes the major factor for DM development as it is responsible for the uncoupling of eNOS, leading to a failure of NO^•^ production and becomes a source of superoxide anion (O_2_^•^ –) [39], [40]. This uncoupling, however, can be reverted by the administration of an antioxidant [41].

At the kidney hystology, the most significant changes were seen in the tubules, including the vacuolization and glycosidic degeneration observed in the renal cortex and medulla. In animals that were supplemented with NAC or the ones who received aerobic training, these alterations were reduced; this protection was increased when both treatments were given together. These findings are similar to unpublished observations about DM, from our laboratory, and they corroborate with reports from other researchers showing that antioxidant treatment [42] or the regular aerobic exercises contributed to reduce the injuries and preserved the renal structure in animals with DM [43].

Given the link between oxidative stress and diabetes, and the strong modulation of P2X_7_ receptor by the oxidative stress, we decided to evaluate its expression [44]. Our findings showed that P2X_7_-R expression was strongly correlated with lipoperoxidation, which is corroborated by others that have demonstrated that this receptor can induce ROS and RNS production [45]. However, there are no studies that show the opposite, in other words, whether redox homeostasis compromise the P2×_7_-R activation is unclear. Recent papers showed that proinflammatory cytokines and hyperglycemia can both act on p38 MAPK mechanisms and increase extracellular ATP levels, resulting in the increased expression of P2X_7_-R [46], [47]. In our study, we observed that exercises and/or NAC administration reduced the P2X_7_-R expression in the kidneys of diabetic animals, suggesting us the interdependence of this receptor to oxidative stress.

The activation of P2X_7_-R increases the permeability of the cell membrane, allowing the transport of many cations, including calcium (Ca^2+^). An increase in cytoplasmic Ca^2+^ may cause cell swelling and cell death by necrosis or apoptosis [48], [49]. A recent study by Bourzac and colleagues[50] reported additional functions of this receptor, and showed that the membrane activity of glucose transporter-2 could be down-regulated by P2X_7_-R activation; thus this receptor can modulate the absorption of glucose and can promote uncontrolled hyperglycemia. When we evaluated Ca^2+^ mobility, diabetic animals had the highest levels of Ca^2+^ influx, reflecting highly activated P2X_7_-R and blood glucose, which were significantly reduced with NAC administration and/or aerobic training.

Our findings are supported by studies that have shown that the influx of Ca^2+^ can be modulated in the intracellular environment, by free radicals and voltage-dependent Ca^2+^ channels. In this case, ROS acts as an agonist, binding to the intracellular terminals of the receptor and opening the pores [51]. On the other hand, a category of free radicals, including reactive nitrogen species (RNS), have been shown to act as antagonists that inhibit Ca^2+^ influx. This effect is believed to be a result of nitrosylation by NO^•^ in the intracellular terminals of the receptor [51], [52].

A study by Coddou and colleagues [53] showed that the P2×_2a_ and P2×_4_ receptors could be modulated by redox balance, and specifically, that extracellular ATP functions as an agonist and that these receptors could be activated by intracellular ROS. These findings emphasize that P2ΣX_7_-R is not only a sensor of extracellular ATP but may also respond to intracellular stimuli that changes depending on the redox homeostasis of the cell.

Our study suggests that the use of strategies that modulate P2X_7_ receptor, such as NAC or aerobic training, especially when both are associated, they result in renoprotective action, reducing diabetic nephropathy.

To our knowledge, this study is the first to show the possible modulation of P2X_7_-R by NO^•^, because we have seen that when oxidative stress is reduced, NO^•^ bioavailability is increased; is this situation NO^•^ could perhaps be inhibiting the receptor through its nitrosylation. We believe that these findings need more investigations to identify the intracellular pathways that would modulate P2×_7_-R activity, and hence this, could provide therapeutic targets to the prevention of diabetic complications.

## References

[1] Molitch ME, DeFronzo RA, Franz MJ, Keane WF, Mogensen CE, et al (2004) Nephropathy in diabetes. Diabetes Care 27 Suppl 1S79–83. 14693934 10.2337/diacare.27.2007.s79

[2] Nogueira GB, Rodrigues AM, Maciel FR, Punaro GR, Mouro MG, et al.. (2011) N-acetylcysteine and oxidative stress in the kidney of uninephrectomized rats with diabetes mellitus. Philadelphia: ASN Kidney Week 2011 Annual Meeting. 1.

[3] Sen CK (2001) Antioxidant and redox regulation of cellular signaling: introduction. Med Sci Sports Exerc 33: 368–370. 11252060 10.1097/00005768-200103000-00005

[4] Ji LL, Katz A, Fu R, Griffiths M, Spencer M (1993) Blood glutathione status during exercise: effect of carbohydrate supplementation. J Appl Physiol 74: 788–792. 8384616 10.1152/jappl.1993.74.2.788

[5] Winnick JJ, Sherman WM, Habash DL, Stout MB, Failla ML, et al (2008) Short-term aerobic exercise training in obese humans with type 2 diabetes mellitus improves whole-body insulin sensitivity through gains in peripheral, not hepatic insulin sensitivity. J Clin Endocrinol Metab 93: 771–778. 18073312 10.1210/jc.2007-1524 PMC2266960

[6] Madden KM (2013) Evidence for the benefit of exercise therapy in patients with type 2 diabetes. Diabetes Metab Syndr Obes 6: 233–239. 23847428 10.2147/DMSO.S32951 PMC3704296

[7] Thaning P, Bune LT, Hellsten Y, Pilegaard H, Saltin B, et al (2010) Attenuated purinergic receptor function in patients with type 2 diabetes. Diabetes 59: 182–189. 19808895 10.2337/db09-1068 PMC2797920

[8] Ralevic V, Burnstock G (1998) Receptors for purines and pyrimidines. Pharmacol Rev 50: 413–492. 9755289

[9] Vonend O, Turner CM, Chan CM, Loesch A, Dell’Anna GC, et al (2004) Glomerular expression of the ATP-sensitive P2X receptor in diabetic and hypertensive rat models. Kidney Int 66: 157–166. 15200422 10.1111/j.1523-1755.2004.00717.x

[10] Punaro GR, Maciel FR, Rodrigues AM, Rogero MM, Bogsan CS, et al.. (2014) Kefir administration reduced progression of renal injury in STZ-diabetic rats by lowering oxidative stress. Nitric Oxide. 10.1016/j.niox.2013.12.012 24406684

[11] Ochodnicky P, de Zeeuw D, Henning RH, Kluppel CA, van Dokkum RP (2006) Endothelial function predicts the development of renal damage after combined nephrectomy and myocardial infarction. J Am Soc Nephrol 17: S49–52. 16565247 10.1681/ASN.2005121322

[12] Roughan JV, Flecknell PA (2004) Behaviour-based assessment of the duration of laparotomy-induced abdominal pain and the analgesic effects of carprofen and buprenorphine in rats. Behav Pharmacol 15: 461–472. 15472568 10.1097/00008877-200411000-00002

[13] Smith JM, Paulson DJ, M Solar S (1997) Na+/K+-ATPase activity in vascular smooth muscle from streptozotocin diabetic rat. Cardiovascular Research 34: 137–144. 9217883 10.1016/s0008-6363(96)00238-6

[14] Rodrigues AM, Bergamaschi CT, Araujo RC, Mouro MG, Rosa TS, et al (2011) Effects of training and nitric oxide on diabetic nephropathy progression in type I diabetic rats. Exp Biol Med (Maywood) 236: 1180–1187. 21930716 10.1258/ebm.2011.011005

[15] Shimizu MH, Coimbra TM, de Araujo M, Menezes LF, Seguro AC (2005) N-acetylcysteine attenuates the progression of chronic renal failure. Kidney Int 68: 2208–2217. 16221220 10.1111/j.1523-1755.2005.00677.x

[16] Mancini G, Carbonara AO, Heremans JF (1965) Immunochemical quantitation of antigens by single radial immunodiffusion. Immunochemistry 2: 235–254. 4956917 10.1016/0019-2791(65)90004-2

[17] Bernheim F, Bernheim ML, Wilbur KM (1948) The reaction between thiobarbituric acid and the oxidation products of certain lipides. J Biol Chem 174: 257–264. 18914082

[18] Tirkey N, Kaur G, Vij G, Chopra K (2005) Curcumin, a diferuloylmethane, attenuates cyclosporine-induced renal dysfunction and oxidative stress in rat kidneys. BMC Pharmacol 5: 15. 16225695 10.1186/1471-2210-5-15 PMC1277828

[19] Manna P, Sinha M, Sil PC (2006) Aqueous extract of Terminalia arjuna prevents carbon tetrachloride induced hepatic and renal disorders. BMC Complement Altern Med 6: 33. 17010209 10.1186/1472-6882-6-33 PMC1599753

[20] Shimizu MH, Danilovic A, Andrade L, Volpini RA, Liborio AB, et al (2008) N-acetylcysteine protects against renal injury following bilateral ureteral obstruction. Nephrol Dial Transplant 23: 3067–3073. 18469310 10.1093/ndt/gfn237 PMC2542407

[21] Leandro SM, Furukawa LN, Shimizu MH, Casarini DE, Seguro AC, et al (2008) Low birth weight in response to salt restriction during pregnancy is not due to alterations in uterine-placental blood flow or the placental and peripheral renin-angiotensin system. Physiol Behav 95: 145–151. 18572207 10.1016/j.physbeh.2008.05.011

[22] Torres LL, Quaglio NB, de Souza GT, Garcia RT, Dati LM, et al (2011) Peripheral oxidative stress biomarkers in mild cognitive impairment and Alzheimer's disease. J Alzheimers Dis 26: 59–68. 21593563 10.3233/JAD-2011-110284

[23] Hampl V, Walters CL, Archer SL (1996) Determination of nitric oxide by the chemiluminescence reaction with ozone. In: Feelisch M, Stamler JS, editors. Methods in nitric oxide research. Chichester: John Wiley & Sons. 310–318.

[24] Doná F, Ulrich H, Persike DS, Conceição IM, Blini JP, et al (2009) Alteration of purinergic P2×4 and P2×7 receptor expression in rats with temporal-lobe epilepsy induced by pilocarpine. Epilepsy Research 83: 157–167. 19084381 10.1016/j.eplepsyres.2008.10.008

[25] Paredes-Gamero EJ, Leon CM, Borojevic R, Oshiro ME, Ferreira AT (2008) Changes in intracellular Ca2+ levels induced by cytokines and P2 agonists differentially modulate proliferation or commitment with macrophage differentiation in murine hematopoietic cells. J Biol Chem 283: 31909–31919. 18775989 10.1074/jbc.M801990200

[26] De Vriese AS, Verbeuren TJ, Van de Voorde J, Lameire NH, Vanhoutte PM (2000) Endothelial dysfunction in diabetes. Br J Pharmacol 130: 963–974. 10882379 10.1038/sj.bjp.0703393 PMC1572156

[27] Shimamoto K, Hayashi H, Taniai E, Morita R, Imaoka M, et al (2011) Antioxidant N-acetyl-L-cysteine (NAC) supplementation reduces reactive oxygen species (ROS)-mediated hepatocellular tumor promotion of indole-3-carbinol (I3C) in rats. J Toxicol Sci 36: 775–786. 22129741 10.2131/jts.36.775

[28] Santin K, da Rocha RF, Cechetti F, Quincozes-Santos A, de Souza DF, et al (2011) Moderate exercise training and chronic caloric restriction modulate redox status in rat hippocampus. Brain Res 1421: 1–10. 21974860 10.1016/j.brainres.2011.08.003

[29] Rocha RE, Coelho I, Pequito DC, Yamagushi A, Borghetti G, et al (2013) Interval training attenuates the metabolic disturbances in type 1 diabetes rat model. Arq Bras Endocrinol Metabol 57: 594–602. 24343627 10.1590/s0004-27302013000800003

[30] Afolayan AJ, Sunmonu TO (2012) Protective role of Artemisia afra aqueous extract on tissue antioxidant defense systems in streptozotocin-induced diabetic rats. Afr J Tradit Complement Altern Med 10: 15–20. 24082320 10.4314/ajtcam.v10i1.3 PMC3746352

[31] da Cunha MJ, da Cunha AA, Ferreira GK, Baladao ME, Savio LE, et al (2013) The effect of exercise on the oxidative stress induced by experimental lung injury. Life Sci 92: 218–227. 23295959 10.1016/j.lfs.2012.12.005

[32] Forbes SC, Little JP, Candow DG (2012) Exercise and nutritional interventions for improving aging muscle health. Endocrine 42: 29–38. 22527891 10.1007/s12020-012-9676-1

[33] Romanelli G, Giustina A, Cravarezza P, Caldonazzo A, Agabiti-Rosei E, et al (1991) Albuminuria induced by exercise in hypertensive type I and type II diabetic patients: a randomised, double-blind study on the effects of acute administration of captopril and nifedipine. J Hum Hypertens 5: 167–173. 1920340

[34] Debevec T, Pialoux V, Mekjavic IB, Eiken O, Mury P, et al.. (2013) Moderate Exercise Blunts Oxidative Stress induced by Normobaric Hypoxic Confinement. Med Sci Sports Exerc. 10.1249/MSS.0b013e31829f87ef 23846158

[35] Conti V, Russomanno G, Corbi G, Guerra G, Grasso C, et al (2013) Aerobic training workload affects human endothelial cells redox homeostasis. Med Sci Sports Exerc 45: 644–653. 23135374 10.1249/MSS.0b013e318279fb59

[36] Cosenzi A, Bernobich E, Bonavita M, Trevisan R, Bellini G, et al (2002) Early effects of diabetes on inducible nitric oxide synthase in the kidney. Acta Diabetol 39: 91–96. 12120919 10.1007/s005920200019

[37] Song W, Kwak HB, Kim JH, Lawler JM (2009) Exercise training modulates the nitric oxide synthase profile in skeletal muscle from old rats. J Gerontol A Biol Sci Med Sci 64: 540–549. 19304939 10.1093/gerona/glp021 PMC2800810

[38] Grutzmacher C, Park S, Zhao Y, Morrison ME, Sheibani N, et al (2013) Aberrant production of extracellular matrix proteins and dysfunction in kidney endothelial cells with a short duration of diabetes. Am J Physiol Renal Physiol 304: F19–30. 23077100 10.1152/ajprenal.00036.2012 PMC3543620

[39] Santhanam AV, d’Uscio LV, Smith LA, Katusic ZS (2012) Uncoupling of eNOS causes superoxide anion production and impairs NO signaling in the cerebral microvessels of hph-1 mice. J Neurochem 122: 1211–1218. 22784235 10.1111/j.1471-4159.2012.07872.x PMC3433644

[40] Hoang HH, Padgham SV, Meininger CJ (2013) L-arginine, tetrahydrobiopterin, nitric oxide and diabetes. Curr Opin Clin Nutr Metab Care 16: 76–82. 23164986 10.1097/MCO.0b013e32835ad1ef

[41] Faria AM, Papadimitriou A, Silva KC, Lopes de Faria JM, Lopes de Faria JB (2012) Uncoupling endothelial nitric oxide synthase is ameliorated by green tea in experimental diabetes by re-establishing tetrahydrobiopterin levels. Diabetes 61: 1838–1847. 22586583 10.2337/db11-1241 PMC3379677

[42] Kukner A, Colakoglu N, Ozogul C, Naziroglu M, Firat T (2009) The effects of combined vitamin C and E in streptozotocin-induced diabetic rat kidney. Clinical Reviews and Opinions 1: 029–036.

[43] Kurdak H, Sandikci S, Ergen N, Dogan A, Kurdak SS (2010) The effects of regular aerobic exercise on renal functions in streptozotocin induced diabetic rats. J Sports Sci Med 9: 294–299. 24149699 PMC3761734

[44] Volonte C, Apolloni S, Skaper SD, Burnstock G (2012) P2×7 receptors: channels, pores and more. CNS Neurol Disord Drug Targets 11: 705–721. 22963440 10.2174/187152712803581137

[45] Hewinson J, Mackenzie AB (2007) P2X(7) receptor-mediated reactive oxygen and nitrogen species formation: from receptor to generators. Biochem Soc Trans 35: 1168–1170. 17956304 10.1042/BST0351168

[46] Xu H, Wu B, Jiang F, Xiong S, Zhang B, et al.. (2013) High fatty acids modulate P2X expression and IL-6 release via the p38 MAPK pathway in PC12 cells. Brain Res Bull. 10.1016/j.brainresbull.2013.02.002 23438872

[47] Trueblood KE, Mohr S, Dubyak GR (2011) Purinergic regulation of high-glucose-induced caspase-1 activation in the rat retinal Muller cell line rMC-1. Am J Physiol Cell Physiol 301: C1213–1223. 21832250 10.1152/ajpcell.00265.2011 PMC3213916

[48] Schulze-Lohoff E, Hugo C, Rost S, Arnold S, Gruber A, et al (1998) Extracellular ATP causes apoptosis and necrosis of cultured mesangial cells via P2Z/P2×7 receptors. Am J Physiol 275: F962–971. 9843914 10.1152/ajprenal.1998.275.6.F962

[49] Souza CO, Santoro GF, Figliuolo VR, Nanini HF, de Souza HS, et al (2012) Extracellular ATP induces cell death in human intestinal epithelial cells. Biochim Biophys Acta 1820: 1867–1878. 22951220 10.1016/j.bbagen.2012.08.013

[50] Bourzac JF, L’Eriger K, Larrivee JF, Arguin G, Bilodeau MS, et al (2013) Glucose transporter 2 expression is down regulated following P2×7 activation in enterocytes. J Cell Physiol 228: 120–129. 22566162 10.1002/jcp.24111

[51] Annunziato L, Pannaccione A, Cataldi M, Secondo A, Castaldo P, et al (2002) Modulation of ion channels by reactive oxygen and nitrogen species: a pathophysiological role in brain aging? Neurobiol Aging 23: 819–834. 12392785 10.1016/s0197-4580(02)00069-6

[52] Summers BA, Overholt JL, Prabhakar NR (1999) Nitric oxide inhibits L-type Ca2+ current in glomus cells of the rabbit carotid body via a cGMP-independent mechanism. J Neurophysiol 81: 1449–1457. 10200181 10.1152/jn.1999.81.4.1449

[53] Coddou C, Codocedo JF, Li S, Lillo JG, Acuna-Castillo C, et al (2009) Reactive oxygen species potentiate the P2×2 receptor activity through intracellular Cys430. J Neurosci 29: 12284–12291. 19793987 10.1523/JNEUROSCI.2096-09.2009 PMC6501587

