# Correction: P2×_7_ Receptor in the Kidneys of Diabetic Rats Submitted to Aerobic Training or to N-Acetylcysteine Supplementation

**DOI:** 10.1371/journal.pone.0110078

**Published:** 2014-09-29

**Authors:** The PLOS ONE Staff

The fifth author’s name is spelled incorrectly. The correct name is Marcus V. Buri. The correct citation is: Rodrigues AM, Bergamaschi CT, Fernandes MJS, Paredes-Gamero EJ, Buri MV, et al. (2014) P2X_7_ Receptor in the Kidneys of Diabetic Rats Submitted to Aerobic Training or to N-Acetylcysteine Supplementation. PLoS ONE 9(6): e97452. doi:10.1371/journal.pone.0097452

There is an error in the article title. The correct title is P2X_7_ Receptor in the Kidneys of Diabetic Rats Submitted to Aerobic Training or to N-Acetylcysteine Supplementation. The correct citation is: Rodrigues AM, Bergamaschi CT, Fernandes MJS, Paredes-Gamero EJ, Buri MV, et al. (2014) P2X_7_ Receptor in the Kidneys of Diabetic Rats Submitted to Aerobic Training or to N-Acetylcysteine Supplementation. PLoS ONE 9(6): e97452. doi:10.1371/journal.pone.0097452
